# Supplementary material for: Interactions of husbandry, landscape, and immunity in regulating viral loads for managed honey bees
Source: Biol Open. 2025 Sep 22;14(9):bio062201. doi: 10.1242/bio.062201 (PMC12505265; doi:10.1242/bio.062201)
Supplement: Supplementary information [file biolopen-14-062201-s1.pdf]

Table S1. Primers used to amplify viral and immune targets for qPCR.

Primer	Sequence (5’-> 3’)	Size (bp)	Accession #	Ref.
DWV_A-F	TTCATTAAAGCCACCTGGAACATC	143	PP766259	[2]
DWV_A-R	TTTCCTCATTAACTGTGTCGTTGA			
BQCV-F	TCCTCAAATCTGGAGCGAAC	184	NC_003784	[3]
BQCV-R	GCCAGACTATGCGACATATCATT			
IAPV-F	CCATGCCTGGCGATTCAC	203	EU436443	[2]
IAPV-R	CTGAATAATACTGTGCGTATC			
LSV_2-F	CGTGCTGAGGCCACGGTTGT	225	OR496467	[3]
LSV_2-R	GCGGTGTCGATCTCGCGGAC			
actin-F	CGTGCCGATAGTATTCTTGC	138	NM_001185145	[1]
actin-R	CCATTGTCAACTACGAGTGC			
ppo-F	AGATGGCATGCATTTGTTGA	293	NM_001011627	[4]
ppo-R	CCACGCTCGTCTTCTTTAGG			
domeless-F	TTGTGCTCCTGAAAATGCTG	141	XM_006567688	[4]
domeless-R	AACCTCCAAATCGCTCTGTG			
dicer-F	AGCAGTAGCTGATTGTGTGGA	223	XM_026446279	*de novo*
dicer-R	TGAAGGATGTGTAAACGCCTGT			
defensin-F	TGCGCTGCTAACTGTCTCAG	80	NM_001011616	[5]
defensin-R	AATGGCACTTAACCGAAACG			

1. Alger, Samantha A., et al. "RNA virus spillover from managed honeybees (Apis mellifera) to wild bumblebees (Bombus spp.)." *PloS one* 14.6 (2019): e0217822.
2. Locke, Barbara, et al. "Acaricide treatment affects viral dynamics in Varroa destructor-infested honey bee colonies via both host physiology and mite control." *Applied and environmental Microbiology* 78.1 (2012): 227-235.
3. Runckel, Charles, et al. "Temporal analysis of the honey bee microbiome reveals four novel viruses and seasonal prevalence of known viruses, Nosema, and Crithidia." *PloS one* 6.6 (2011): e20656.
4. Evans, Jay D., et al. "Immune pathways and defence mechanisms in honey bees Apis mellifera." *Insect molecular biology* 15.5 (2006): 645-656.
5. Zhao, Yazhou, et al. "The dynamics of deformed wing virus concentration and host defensive gene expression after Varroa mite parasitism in honey bees, Apis mellifera." *Insects* 10.1 (2019): 16.

Table S2. gBlock sequence used for absolute quantification. Ten random base pairs flank each amplicon. Colors refer to the target gene (yellow = BQCV[1]; blue = DWV[2]; red= LSV[3]; green = *Apis mellifera* mRNA actin[4]).

TTTTTTTTTTTTTAGAGCGAATTCGGAAACATTTTACTATAGTTCAGGTCGGAATAATC
TCGATATAGCCACTTCACCTCCTTCCATCAATCGCTACTATGCGGTAGGTGCGGGAG
ATGATATGGACTTTTCCATCTTTATCGGTACGCCTTTTTTTTTTCCTGGACAAGGTCTC
GGTAGAAGGAACGACGAATTGGACTCATGCTCGAGGATTGGGTCGTCGTACAGCAA
CCATAGAAAATGCTAAACAAGCGTTAGAGTTAGCATTTGGGTGGGGTCCTGAATTTT
TTTTTTTTTATCTCGCGCCGCCACCTCATCGTTTGTCGAATCGTCGCTGTTGTCCTTGT
TACGATTTATGCAACAAGTACCTGCAGTTGAGTCCTCTGCAGAAACTCGCCGGTGTC
TTTTATTTGGTGGCCCTAGAGAAAGATTTAAGGGATACGACACCTTTGACCTTGGAT
TCATGGGTGTCGCGGTACCCTCTTTTTTTTTTTCAAAGACCCAAGCTCCCTAACTTGT
ACATAACTTTTTTTTTATATAAAACGAAAAACATAAACCAATAAGCCAACATGTCTG
ACGAAGAAGTTGCAC TTTTTTTTTT

1. Traynor, Kirsten S., et al. "Multiyear survey targeting disease incidence in US honey bees." Apidologie 47 (2016): 325-347.
2. Chantawannakul, P., et al. "A scientific note on the detection of honeybee viruses using real-time PCR (TaqMan) in Varroa mites collected from a Thai honeybee (Apis mellifera) apiary." Journal of invertebrate pathology 91.1 (2006): 69-73.
3. Runckel, Charles, et al. "Temporal analysis of the honey bee microbiome reveals four novel viruses and seasonal prevalence of known viruses, Nosema, and Crithidia." PloS one 6.6 (2011): e20656.
4. Elsik, Christine G., et al. "Finding the missing honey bee genes: lessons learned from a genome upgrade." BMC genomics 15 (2014): 1-29.

Biology Open • Supplementary information

Table S3. Associations among husbandry interventions indicating correlated use patterns. ρ = Spearman rank correlation coefficient.

Var1	Var2	ρ	P
SHB	SF	0.07	0.2623
EO	SF	-0.01	0.8792
EO	SHB	0.07	0.266
Api	SF	0.25	0.0001
Api	SHB	0.05	0.4429
Api	EO	-0.04	0.5555
Ami	SF	-0.08	0.2385
Ami	SHB	-0.18	0.0056
Ami	EO	-0.15	0.0185
Ami	Api	0.02	0.767
Hops	SF	0.24	0.0002
Hops	SHB	0.17	0.0077
Hops	EO	0.00	0.9546
Hops	Api	-0.12	0.0574
Hops	Ami	-0.20	0.0017
OA	SF	-0.19	0.0035
OA	SHB	0.18	0.0046
OA	EO	-0.19	0.0024
OA	Api	-0.02	0.8159
OA	Ami	0.39	<.0001
OA	Hops	-0.09	0.1792
FA	SF	-0.02	0.8067
FA	SHB	0.02	0.7654
FA	EO	0.14	0.0326
FA	Api	-0.13	0.0453
FA	Ami	0.05	0.4533
FA	Hops	0.20	0.0014
FA	OA	0.03	0.6253

Biology Open • Supplementary information

Table S4. Correlations within immunity and landscape variables. ρ = Spearman rank correlation coefficient.

Var1	Var2	ρ	P
**A. Landscape variables**			
IS	BFA	-0.80	<.0001
NL	BFA	0.85	<.0001
NL	IS	-0.87	<.0001
Ag	BFA	0.76	<.0001
Ag	IS	-0.90	<.0001
Ag	NL	0.77	<.0001
OW	BFA	-0.06	0.3265
OW	IS	-0.12	0.0579
OW	NL	-0.11	0.0888
OW	Ag	0.07	0.2496
**B. Immune genes**			
def	*ppo*	0.50	<.0001
dome	*ppo*	0.88	<.0001
dome	*def*	0.56	<.0001
dicer	*ppo*	0.62	<.0001
dicer	*def*	0.46	<.0001
dicer	*dome*	0.80	<.0001

Biology Open • Supplementary information
